# Supplementary material for: Nuclease genes occupy boundaries of genetic exchange between bacteriophages
Source: NAR Genom Bioinform. 2023 Aug 24;5(3):lqad076. doi: 10.1093/nargab/lqad076 (PMC10448857; doi:10.1093/nargab/lqad076)
Supplement: lqad076_Supplemental_Files [file lqad076_supplemental_files.zip › Figs S1-S4 and Tables S1-S3.pdf]

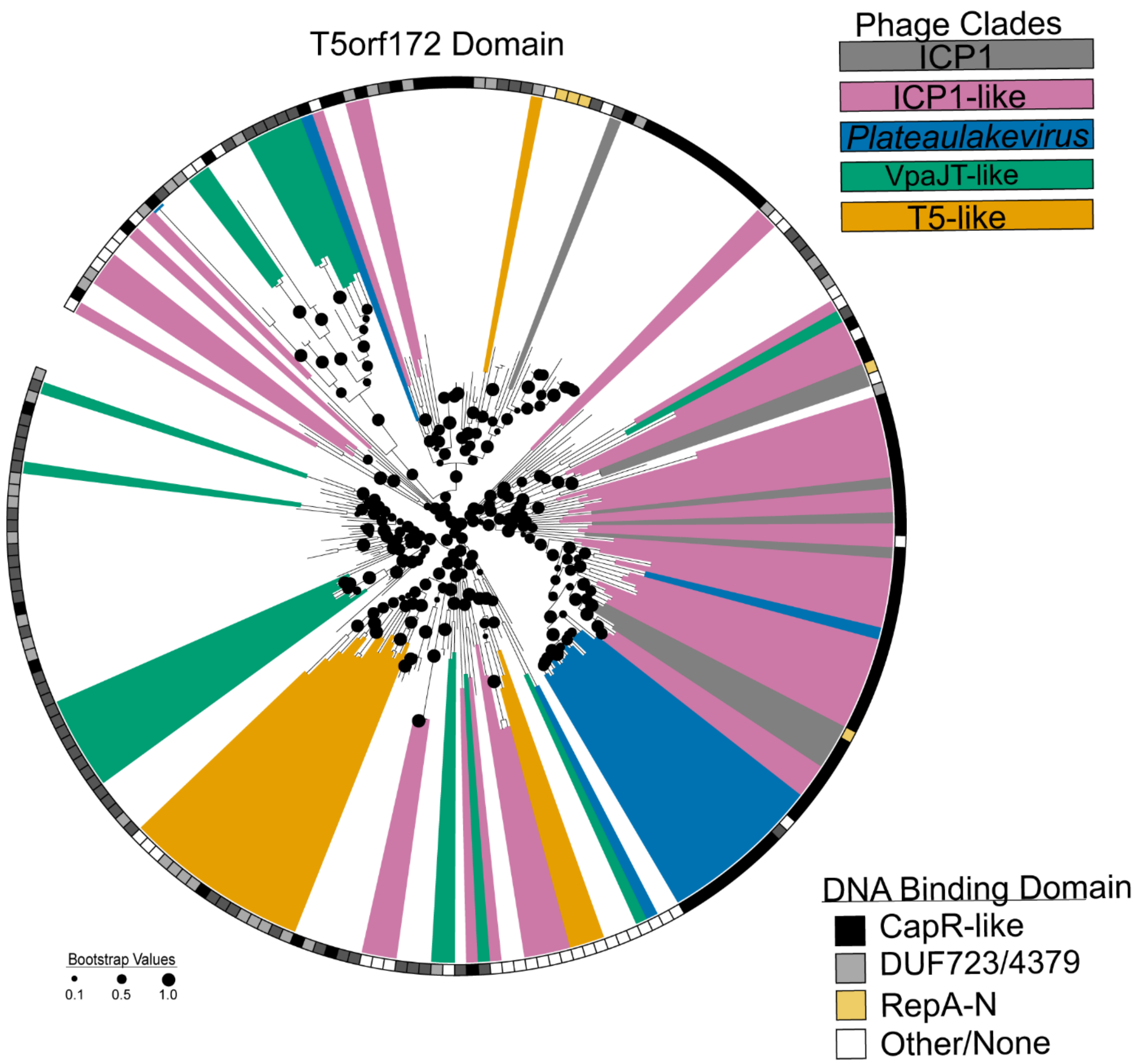

Figure S1. Phylogenetic analysis of the distinct T5orf172 domains of phages
Alignment of the T5orf172 domains from each of the identified T5orf172 HEG-containing phages. Phage clades are determined based on the phylogenetic tree in Figure 2. The ring of boxes surrounding the tree corresponds to the identified DNA binding domain for each individually graphed HEG, as shown in Figure 5. Bootstrap values are indicated by the black circles, scaled according to the legend in the bottom left.

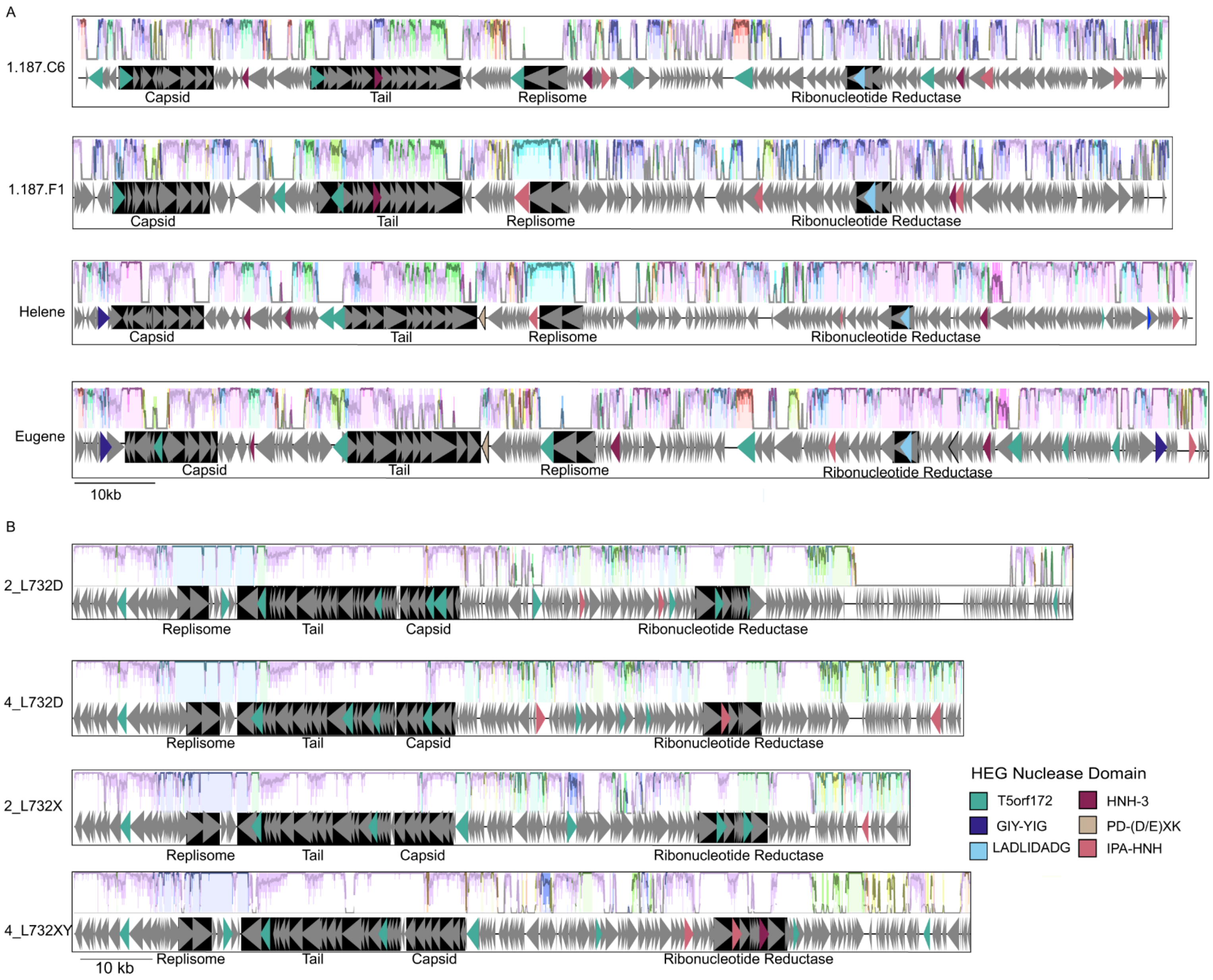
A
1.187.C6
Capsid
Tail
Replisome
Ribonucleotide Reductase
1.187.F1
Capsid
Tail
Replisome
Ribonucleotide Reductase
Helene
Capsid
Tail
Replisome
Ribonucleotide Reductase
Eugene
Capsid
Tail
Replisome
Ribonucleotide Reductase
10kb
B
2_L732D
Replisome
Tail
Capsid
Ribonucleotide Reductase
4_L732D
Replisome
Tail
Capsid
Ribonucleotide Reductase
2_L732X
Replisome
Tail
Capsid
Ribonucleotide Reductase
4_L732XY
Replisome
Tail
Capsid
Ribonucleotide Reductase
10 kb
HEG Nuclease Domain
T5orf172
HNH-3
GIY-YIG
PD-(D/E)XK
LADLIDADG
IPA-HNH

Figure S2. Related HEG-Islands containing cargo genes are flanked by variable HEG families in related phages

Whole-genome alignment of ICP1-like (A) and *Plateaulikevirus* (B) generated with Mauve shows the relatedness of HEG-islands in otherwise distinct phages. Genes encoding predicted HEG nuclease domains are colored according to the key, and operons encoding key marker genes for HEG islands are shaded in black. Despite sharing conserved cargo genes, many gene neighborhoods are flanked by HEGs with different nuclease domains.

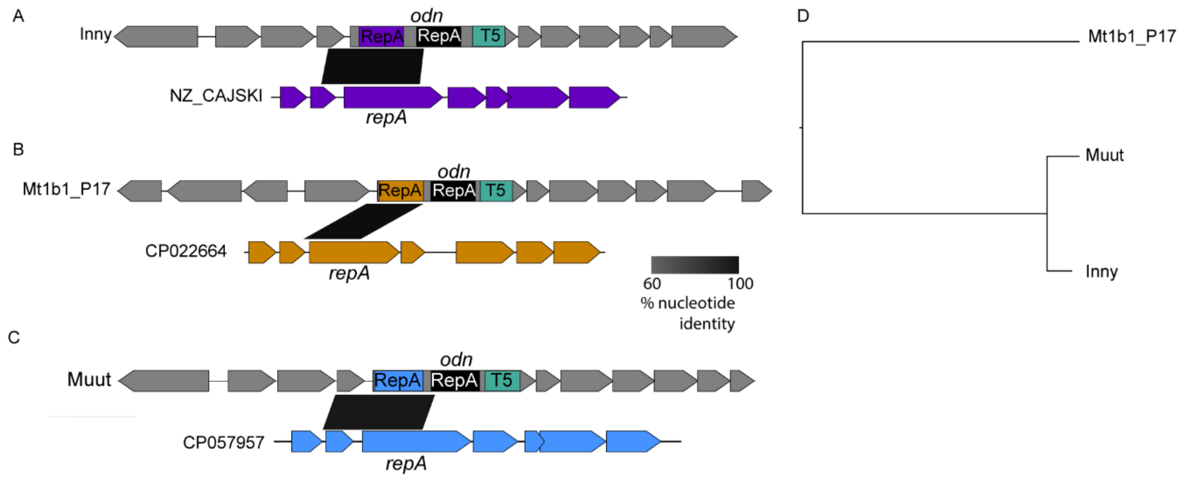

Figure S3. Recombination of HEG-associated domains between phages and their satellites.

(A-C) Examples of recombination between *E. coli* phages shown in Figure 8 and their satellites, specifically highlighting the RepA-N domain homology of the satellites to the respective phages. Regions of > 60% identity are marked according to the legend with black parallelograms.

(D) VipTreeGen phylogeny of each of the three phage genomes, showing the variable conservation of coding sequences between the phages

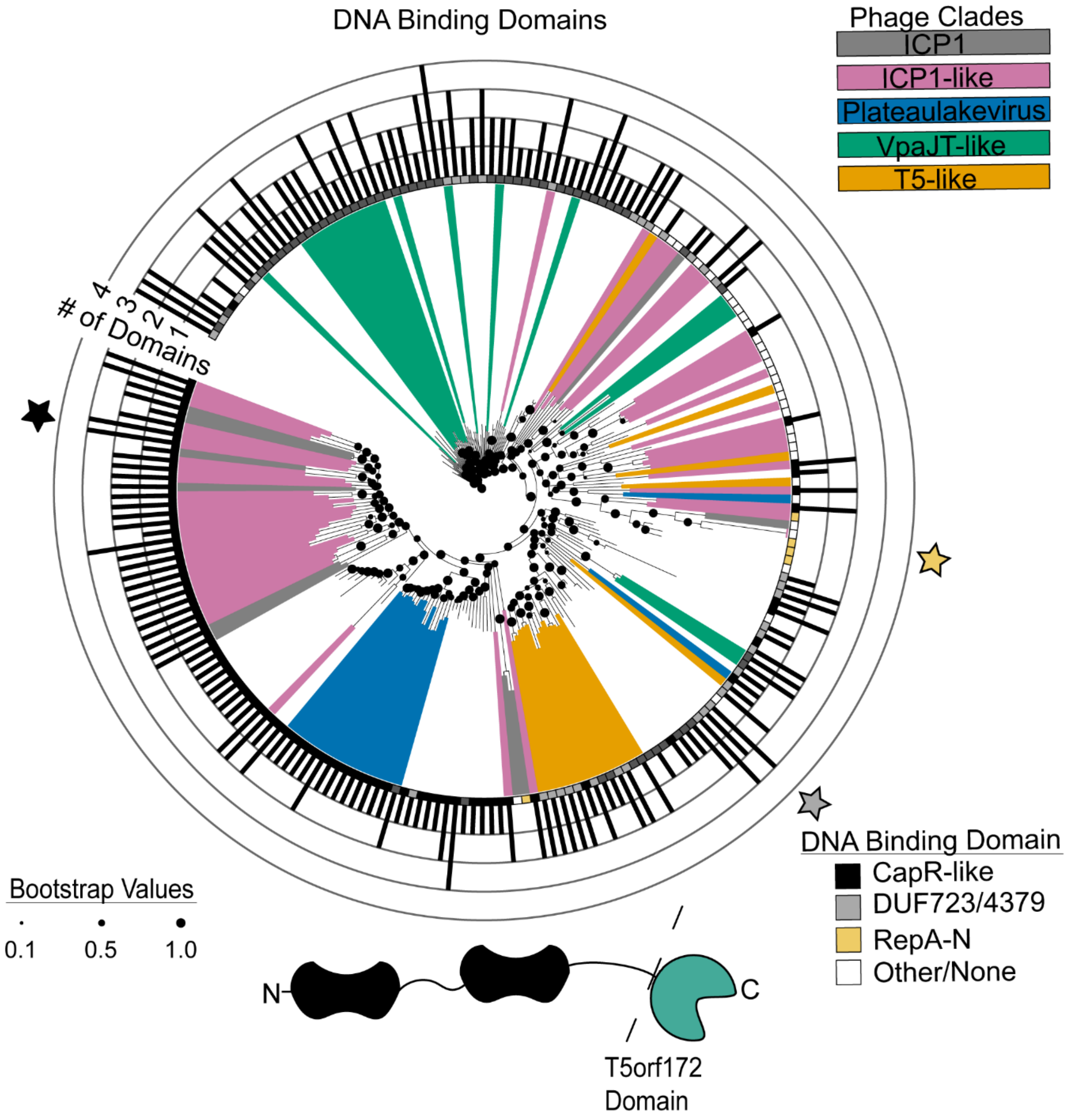

Figure S4.

T5orf172 HEGs regularly contain repeated DNA Binding domains from related Zn-finger domains.

Alignment of the amino acid sequence of the DNA binding domain(s) of each T5orf172 HEG shows the relationship between the domains. After truncating the amino acid sequence of each T5orf172 HEG to remove the T5orf172 domain, as represented by the dashed line in the model below the figure, the remaining sequence was searched for CapR DNA binding and highly related DUF723 or DUF4397 domains with hmmsearch

and boxes at leaf tips were labeled accordingly. The number of DNA binding domains identified by hmmsearch are indicated by the bar graph surrounding the tree. If none of the three Zn-finger domains were identified, the sequence was searched with HHsearch to detect DNA binding domains. Domains identified by HHsearch do not have the number of domains quantified, resulting in a filled-in outer square without a domain occurrence count. Colored stars indicate the representative examples shown in (B), colored according to DNA binding domain type. Supplementary to Figure 5, with bootstrap values added to the supplementary file according to the legend in the bottom left.

Table S1) *Vibrio cholerae* and ICP1 Isolates used in this study
Accession information for genomes of *Vibrio cholerae* and ICP1 references used throughout this study

**Strain**	**Accession**	**Genome Citation**
Vibrio cholerae E7946	CP024162, CP024163	Camilli, A. 2017
ICP1 1992_Ind_M4	MW794141	Boyd et al., 2021
ICP1 2001_Dha_0	HQ641347	Seed et al., 2011
ICP1 2001_Dha_A	HQ641353	Seed et al., 2011
ICP1 2003_Dha_A	MW794140	Boyd et al., 2021
ICP1 2004_Dha_A	HQ641354	Seed et al., 2011
ICP1 2005_Dha_A	HQ641352	Seed et al., 2011
ICP1 2006_Dha_A	HQ641351	Seed et al., 2011
ICP1 2006_Dha_B	HQ641350	Seed et al., 2011
ICP1 2006_Dha_C	HQ641349	Seed et al., 2011
ICP1 2006_Dha_D	HQ641348	Seed et al., 2011
ICP1 2006_Dha_E	MH310934	Angermeyer et al., 2018
ICP1 2006_Dha_EΔCRΔCas2_3	N/A	McKitterick and Seed, 2018
ICP1 2011_Dha_A	MH310933	Angermeyer et al., 2018
ICP1 2011_Dha_B	MH310935	Angermeyer et al., 2018
ICP1 2012_Ind_A	MH310936	Angermeyer et al., 2018
ICP1 2015_Dha_A	MW794150	Boyd et al., 2021
ICP1 2016_Dha_A	MW794151	LeGault et al., 2021
ICP1 2017_Dha_A	MW794152	LeGault et al., 2021
ICP1 2017_Dha_AA	MW794153	LeGault et al., 2021
ICP1 2017_Dha_AB	MW794154	LeGault et al., 2021
ICP1 2017_Dha_AC	MW794155	LeGault et al., 2021
ICP1 2017_Dha_AD	MW794156	LeGault et al., 2021
ICP1 2017_Dha_AE	MW794157	LeGault et al., 2021
ICP1 2017_Dha_B	MW794158	LeGault et al., 2021
ICP1 2017_Dha_C	MW794159	LeGault et al., 2021
ICP1 2017_Dha_D	MW794160	LeGault et al., 2021
ICP1 2017_Dha_E	MW794161	LeGault et al., 2021
ICP1 2017_Dha_F	MN419153	LeGault et al., 2021
ICP1 2017_Dha_N	MW794162	LeGault et al., 2021
ICP1 2017_Dha_O	MW794163	LeGault et al., 2021
ICP1 2017_Dha_P	MW794164	LeGault et al., 2021
ICP1 2017_Dha_R	MW794165	LeGault et al., 2021
ICP1 2017_Dha_S	MW794166	LeGault et al., 2021
ICP1 2017_Dha_V	MW794167	LeGault et al., 2021

ICP1 2017_Dha_W	MW794168	LeGault et al., 2021
ICP1 2017_Dha_X	MW794169	LeGault et al., 2021
ICP1 2017_Dha_Y	MW794170	LeGault et al., 2021
ICP1 2017_Dha_Z	MW794171	LeGault et al., 2021
ICP1 2017_DRC_106	MW794142	Alam et al., 2022
ICP1 2017_DRC_32	MW794143	Alam et al., 2022
ICP1 2017_DRC_48	MW794144	Alam et al., 2022
ICP1 2017_DRC_55	MW794145	Alam et al., 2022
ICP1 2017_DRC_72	MW794146	Alam et al., 2022
ICP1 2017_DRC_74	MW794147	Alam et al., 2022
ICP1 2017_DRC_82	MW794148	Alam et al., 2022
ICP1 2017_DRC_87	MW794149	Alam et al., 2022
ICP1 2017_Mat_H	MN419153	LeGault et al., 2021
ICP1 2017_Mat_I	MW794172	LeGault et al., 2021
ICP1 2017_Mat_K	MW794173	LeGault et al., 2021
ICP1 2018_Mat_001	MW794174	LeGault et al., 2021
ICP1 2018_Mat_002	MW794175	LeGault et al., 2021
ICP1 2018_Mat_004	MW794176	LeGault et al., 2021
ICP1 2018_Mat_159	MW794177	LeGault et al., 2021
ICP1 2018_Mat_160	MW794178	LeGault et al., 2021
ICP1 2018_Mat_164	MW794179	LeGault et al., 2021
ICP1 2018_Mat_166	MW794180	LeGault et al., 2021
ICP1 2018_Mat_167	MW794181	LeGault et al., 2021
ICP1 2018_Mat_170	MW794182	LeGault et al., 2021
ICP1 2018_Mat_B	MW794183	LeGault et al., 2021
ICP1 2019_Dha_007	MW794184	LeGault et al., 2021
ICP1 2019_Dha_G	MW794185	LeGault et al., 2021
ICP1 2019_Dha_H	MW794186	LeGault et al., 2021
ICP1 2019_Dha_I	MW794187	LeGault et al., 2021
ICP1 2019_Mat_005	MW794188	LeGault et al., 2021
ICP1 2019_Mat_B	MW794189	LeGault et al., 2021
ICP1 2019_Mat_C	MW794190	LeGault et al., 2021
ICP1 2019_Mat_D	MW794191	LeGault et al., 2021
ICP1 2019_Mat_E	MW794192	LeGault et al., 2021

Table S2) Other organisms referenced in this study
The accession numbers and shorthand notation for all strains referred to in the study.

Accession	Shortened name[a]	Common name	Host	Genome Citation
AP014858	RYC	Vibrio phage RYC	*Vibrio coralliilyticus*	Ramphul et al. 2017
HQ316579	Helene	Vibrio phage helene 12B3	*Vibrio splendidus*	NA[b]
HQ634156	PWH3a-P1	Vibrio phage PWH3a-P1	*Vibrio natriegens*	NA
HQ634195	Eugene	Vibrio phage eugene 12A10	*Vibrio spp.*	NA
KX507046	S4-7	Vibrio phage S4-7	*Vibrio anguillarum*	NA
NC_047839	SL20	Pseudoalteromonas phage SL20	*Pseudoaltero monas spp.*	NA
NC_048769	2_L372D	Aeromonas phage 2-L372D	*Aeromonas hydrophila*	NA
NC_048770	2_L372X	Aeromonas phage 2-L372X	*Aeromonas hydrophila*	NA
NC_048771	4_L372D	Aeromonas phage 4_L372D	*Aeromonas hydrophila*	NA
NC_048772	4_L372XY	Aeromonas phage 4_L372XY	*Aeromonas hydrophila*	NA
MG592529	1.161..C5	Vibrio phage 1.161.O._10N.261.48.C5	*Vibrio lentus*	Kauffman et al. 2018
MG592562	1.193..C6	Vibrio phage 1.193.O._10N.286.52.C6	*Vibrio splendidus*	Kauffman et al. 2018
MG592473	1.101..C6	Vibrio phage 1.101.O._10N.261.45.C6	*Enterovibrio norvegicus*	Kauffman et al. 2018
MG592553	1.187..F1	Vibrio phage 1.187.O._10N.286.49.F1	*Vibrio splendidus*	Kauffman et al. 2018
AP018813	T2	Enterobacteria phage T2	*Escherichia coli*	Akiyama et al. 2018
NC_000866	T4	Enterobacteria phage T4	*Escherichia coli*	Miller et al. 2003
NC_020843	11895-B1	Vibrio phage 11895-B1	*Vibrio spp.*	NA
NC_025436	1/4	Shewanella sp. phage 1/4	*Shewanella spp.*	Senčilo et al. 2015
NC_025470	1/40	Shewanella sp. phage 1/40	*Shewanella spp.*	Senčilo et al. 2015
NC_029057	qdvp001	Vibrio phage qdvp001	*Vibrio spp.*	NA
MK719750	Barba31A	Rheinheimera phage vB_RspM_Barba31A	*Rheinheimer a spp.*	Nilson et al. 2019
MK719708	Barba4S	Rheinheimera phage vB_RspM_Barba4S	*Rheinheimer a spp.*	Nilson et al. 2019
NC_030934	PsyM_Kil1	Pseudomonas phage vB_PsyM_KIL1	*Pseudomona s syringae*	Rombouts et al. 2016
NC_052657	Muut	Escherichia phage muut	*Escherichia coli*	Olsen et al. 2020

MN850601	Inny	Escherichia phage inny	*Escherichia coli*	Olsen et al. 2020
NC_052662	Mt1B1_P17	Escherichia phage Mt1B1_P17	*Escherichia coli*	NA
CP022664	CP022664	Escherichia coli strain FORC 064 chromosome	Bacterial	NA
NZ_CAJSKI 010000035	NZ_CAJSKI	Escherichia coli isolate Fecal samples	Bacterial	NA
NZ_LBFX01 000018	YB2A06	Vibrio cholerae strain YB2A06	Bacterial	NA
CWPX0100 0013	PLE7 *V. cholerae*	Vibrio cholerae genome assembly 4056_7#9, scaffold ERS013187SCcontig000013	Bacterial	NA
CP057957	N/A	Escherichia coli strain RHB08-C21 chromosome	Bacterial	AbuOun et at. 2021
MH925092	VpaJT	Vibrio phage VpaJT_1	Bacterial	Chen et al. 2019

[a]Phage names shortened for simplicity in figure and text.

[b]Abbreviation: NA, not applicable.

Table S3. Pfam domains used for gene neighborhood analysis

Family	Pfam Domain(s)
Capsid	PF03864, PF05065, PF05357, PF07068
Terminase	PF03592,PF03237,PF04466,PF07471,PF11053,PF16677,PF17288,PF17289,PF05876,PF05944,PF06056,PF03354,PF05119
Tape Measure	PF05017, PF06120, PF06791, PF09718, PF10145, PF20155, PF16459, PF16460, PF16461,PF17388,PF19268
Ribonucleotide Reductase	PF14597,PF00268, PF00317,PF02867,PF08343
DNA Polymerase	PF00476, PF02767
T5orf172	PF10544,PF13455
GIY-YIG	PF01541
HNH-3	PF01381
LAGLIDADG	PF14528,PF03161,PF00961
CapR	Custom profile
IPA-HNH	Custom profile
